# The Quantified Characterization Method of the Micro-Macro Contacts of Three-Dimensional Granular Materials on the Basis of Graph Theory

**DOI:** 10.3390/ma10080898

**Published:** 2017-08-03

**Authors:** Yanpeng Guan, Enzhi Wang, Xiaoli Liu, Sijing Wang, Hebing Luan

**Affiliations:** 1 The State Key Laboratory of Hydro-Science and Engineering, Tsinghua University, Beijing 100084, China; hbgyp2008@163.com (Y.G.); nzwang@tsinghua.edu.cn (E.W.); wangsijing@tsinghua.edu.cn (S.W.); ausluanking@hotmail.com.(H.L.); 2 Sanjiangyuan Collaborative Innovation Center, Tsinghua University, Beijing 100084, China

**Keywords:** granular material, particle contact, microstructure, characterization

## Abstract

We have attempted a multiscale and quantified characterization method of the contact in three-dimensional granular material made of spherical particles, particularly in cemented granular material. Particle contact is defined as a type of surface contact with voids in its surroundings, rather than a point contact. Macro contact is a particle contact set satisfying the restrictive condition of a two-dimensional manifold with a boundary. On the basis of graph theory, two dual geometrical systems are abstracted from the granular pack. The face and the face set, which satisfies the two-dimensional manifold with a boundary in the solid cell system, are extracted to characterize the particle contact and the macro contact, respectively. This characterization method is utilized to improve the post-processing in DEM (Discrete Element Method) from a micro perspective to describe the macro effect of the cemented granular material made of spherical particles. Since the crack has the same shape as its corresponding contact, this method is adopted to characterize the crack and realize its visualization. The integral failure route of the sample can be determined by a graph theory algorithm. The contact force is assigned to the weight value of the face characterizing the particle contact. Since the force vectors can be added, the macro contact force can be solved by adding the weight of its corresponding faces.

## 1. Introduction

The microscopic-macroscopic relationship of the granular material is complex, and the microstructure in the granular material has a great influence on its macroscopic characteristics. Scholars have researched the micro-macro relation of the granular material in different ways and some achievements have been made [1,2,3,4,5,6]. 

Researchers have done much work on the topology of granular systems. Most studies have been concerned with pore fluid transport. Adler et al. reconstructed a number of porous samples and computed their permeability [1,2]. Prasad and Jernot simulated the three-dimensional flow through a porous medium [7]; Lindquist et al. analyzed the void structure in three-dimensional tomographic images of porous media [8]; and Liang et al. related macroscopic transport coefficients to the geometry and topology of the pore space [2]. Hilpert et al. observed the hysteresis in a biconical pore segment [9]. Schroeder-Turk calculated the Minkowski tensor shape of granular structures [10].

The two dual geometrical systems are a good tool to observe the internal structure in the granular material. It can divide the packing space into a large number of solid cells or void cells, respectively [3,4]. Bagi deduced the stress tensor and strain tensor of the granular material by constructing these systems. Li and Li developed a new method to construct the two dual geometrical systems, which was then used to observe the anisotropic behavior of granular materials [5]. Satake put forward a method to create systems fit for the three dimensional granular material and purposed a new definition for the stress and strain [6].

On the other hand, the discrete element method (DEM) is an efficient method to simulate the microscopic-macroscopic relationship of the particulate matter by computing the motion of many small particles [11]. DEM has been used to obtain the microscopic-macroscopic properties of particulate matter [12,13,14]. The topological relation between the components in granular material can improve the progress in DEM. Diederichs hypothesized that the length of the micro-crack is equal to the average value of the diameters of the two parent particles in the two sides of the micro-crack [15]. Zhang and Wong reported that discrete micro-cracks that were close to each other could be artificially connected to form a continuous crack path [16]. These studies have helped to describe the relationship between the macro crack and micro crack of the cemented granular specimen under loads. However, the methods are mostly adjustments for the visualizations provided by DEM, while there remains a lack of mathematical discussions concerning the physics.

The aim of this paper is to put forward a reasonable method to characterize the micro-macro contact of granular material made of spherical particles, particularly the cemented granular material. The structure of this paper is as follows: In Section 2, the micro and macro scales are defined. In Section 3, on the basis of graph theory, the definition of micro contact, and its characterization method are discussed. In Section 4, the characterization method of macro contact is put forward. Taking a rock slope simulated by cemented spherical particles in DEM as an example, Section 5 shows how it applies to the post-processing of DEM. Section 6 discusses the major findings of this paper and outlines our the conclusions.

## 2. The Definition of the Contact

**Figure 1:**
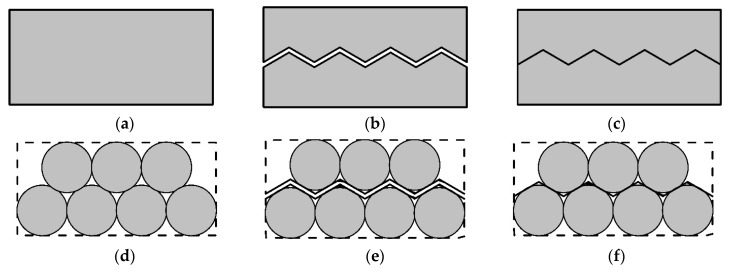
the shape (**a**); the crack (**b**); the contact (**c**) of a cemented solid material; and the shape (**d**); the crack (**e**); the contact (**f**) of a cemented granular material.

**Figure 2:**
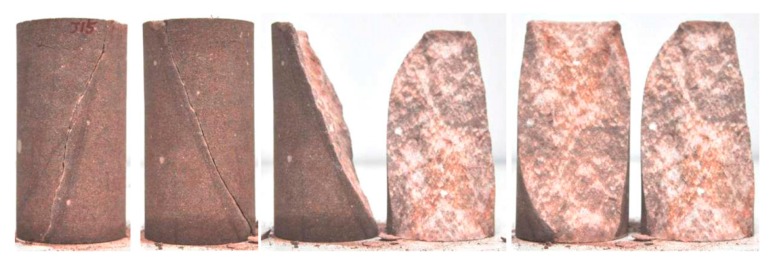
A typical macro contact of granular material [17].

As shown in Figure 1a, this paper employs a common two-dimensional solid material as an example to discuss the contact and crack of granular materials. Figure 2b is a typical crack in a solid material. For each crack, there is always a contact surface corresponding to it, as shown in Figure 2c. Moreover, the crack and its corresponding contact have the same shape.

As shown in Figure 1d, the granular material, particularly the cemented granular material, is a kind of solid material. Likewise, from the macro scale, there may be a crack in the cemented granular material (Figure 1e) and there is always a contact surface corresponding to this crack (Figure 1f). The crack and its corresponding contact surface have the same shape. For the granular material which is not cemented, there are still many contact surfaces inside it. Since its shape is not fixed, the shape of the contact will change with the shape of this material.

This article follows the definition of other researchers with respect to scale [3,4,5]. The particle scale is the microscopic scale. It is assumed that the material in the granular material is not infinitely separable. A particle is the smallest unit of the particulate material. Particle contact is the contact separating particles into two sides, which is a synonym for microscopic contact. For cemented granular materials, if the bonding of two particles is broken, then microscopic cracks will occur at the contact between those two particles.

The laboratory scale or engineering scale is the macroscopic scale. Figure 2 depicts a broken rock sample with internal cemented particles [17]. The rough damaged surface in Figure 2 is a typical macroscopic contact surface inside the cemented particulate material, because it divides all the particles on both sides rather than through the particles. When the bonds at the macroscopic contact are broken, there forms a macro crack at the macroscopic contact.

## 3. Characterization Method of Particle Contact 

### 3.1. The Void Cell System 

There are two dual geometrical systems in granular packs [3,6]: the void cell system G and the solid cell system $G^{\prime }$. The void cell system G=(V, E) is created by the tetrahedralization of particle centers. V is a set of vertexes v which are composed of particle centers.  E is a set of edges e which are composed of the connection of the adjacent particle centers. In this system, each face f is a triangle face formed by three adjacent edges, and each body b is a tetrahedron formed by four adjacent triangle faces.

**Figure 3:**
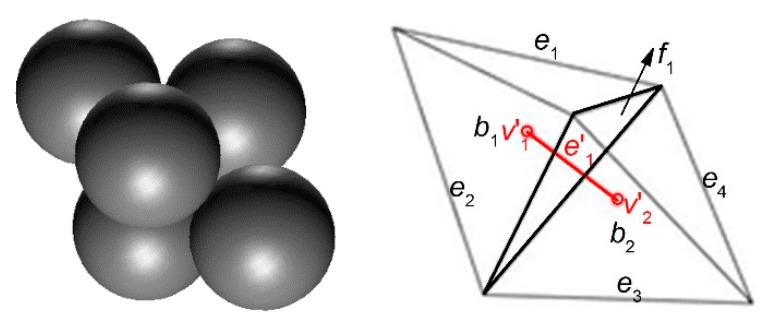
The pack of five particles (**left**) and its two dual geometrical systems (**right**). (v,e,f,b are the vertex, the edge, the face, and the body in the void cell system, respectively. $v^{\prime },e^{\prime },f^{\prime },b^{\prime }$ are the vertex, the edge, the face, and the body in the solid cell system, respectively.)

**Figure 4:**
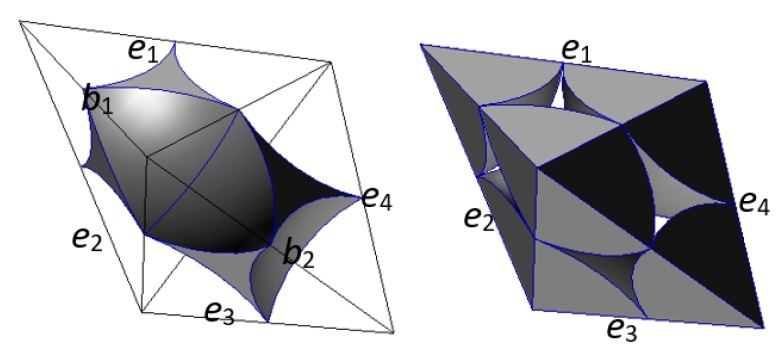
The void part (**left**) and solid part (**right**) in the void cell system. (e,b are the edge and the body in the void cell system, respectively).

The void cell system G can divide the packing space of the granular pack into a set of non-overlapping tetrahedrons. As is shown in Figure 3, five particles are placed compactly and their void cell system G is shown by black lines. The void part and solid part in the tetrahedrons is shown in Figure 4. There are two bodies ($b_{1}$ and $b_{2})$ in this void cell system, and there is an intact void in each body (Figure 4 left). Therefore, each tetrahedron is called a void cell. The face $f_{1}$ is the interface of two adjacent void cells ($b_{1}$ and $b_{2})$.

In the void cell system, the vertex v characterizes the particle. The edge e is the connecting line of two adjacent particles, so it can characterize the particle connection. If these two particles are in contact, the edge e characterizes a particle contact (physical contact). If these two particles are not in contact, the edge e characterizes a virtual contact (the virtual contact is the connection of the centers of the adjacent particles which are not in contact). The face f is the interface of the voids of two adjacent void cells. Therefore, it can characterize the seepage path section. Each body b in this system is a tetrahedron characterizing the void.

### 3.2. The Solid Cell System

The other geometrical system is the solid cell system $G^{\prime }=\left(V^{\prime },\;E^{\prime }\right)$, which is created by the radical Voronoi tessellation of the particle centers [18,19,20]. $V^{\prime }$ is a vertex set which is composed of the centers of the voids in the void cell system G. $E^{\prime }$ is an edge set which is composed of the connection of the void centers. In this system, several edges $e^{\prime }$ aligned end-to-end form a polygon face $f^{\prime }$ and the adjacent faces can form a body $b^{\prime }$.

The void cell system and solid cell system are the dual systems. In the two dual systems, there is a one-to-one relationship between the vertex, edge, face, and body of one system and the body, face, edge, and vertex of the other system, respectively. For example, the body b of the void cell system is a void cell while the vertex $v^{\prime }$ of the solid cell system is the center of the void. Both of them can characterize the void.

As shown by the red vertices and the red line in Figure 3, vertex $v_{1}^{\prime }$ and $v_{2}^{\prime }$ in the solid cell system are the centers of body $b_{1}$ and $b_{2}$ in the void cell system, respectively. The edge $e_{1}^{\prime }$ in the solid cell system characterizes the connection of the void centers ($v_{1}^{\prime }$ and $v_{2}^{\prime }$), so it can characterize the seepage path while the face $f_{1}$ of the void cell system is the seepage path section. Both of them can characterize the seepage path.

**Figure 5:**
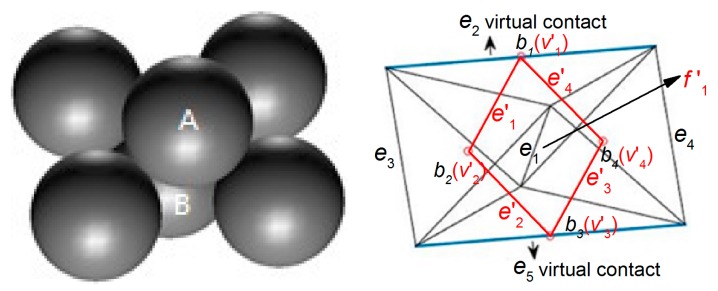
The pack of six particles (**left**) and its two dual geometrical systems (**right**). (v,e,f,b are the vertex, the edge, the face, and the body in the void cell system, respectively. $v^{\prime },e^{\prime },f^{\prime },b^{\prime }$ are the vertex, the edge, the face, and the body in the solid cell system, respectively.)

As shown in Figure 5, six particles are placed compactly and their two dual geometrical systems are shown as black and red lines. In the void cell system G, there are two edges ($e_{2}$ and $e_{5}$) characterizing the virtual contact. The role of the virtual contact is to help divide the packing space into a set of non-overlapping tetrahedrons.

**Figure 6:**
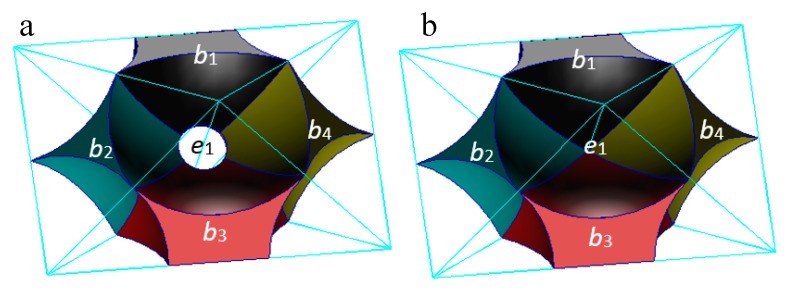
(**a**) Particles A and B are in contact and squeeze each other; (**b**) Particles A and B are not in contact, characterizing the void part in the void cell system. (e,b are the edge and the body in the void cell system, respectively.)

There are four shares of void cells ($b_{1},b_{2},b_{3}$, and $b_{4}$) surrounding the connection between particle A and particle B (edge $e_{1}$) and their centers are $v_{1}^{\prime },\;v_{2}^{\prime },\;v_{3}^{\prime }$, and $v_{4}^{\prime }$ in the solid cell system, respectively. Edges $e_{1}^{\prime },\;e_{2}^{\prime },\;e_{3}^{\prime }$, and $e_{4}^{\prime }$ are the edges joining the vertexes $v_{1}^{\prime },\;v_{2}^{\prime },\;v_{3}^{\prime }$, and $v_{4}^{\prime }$. In physics, the macro contact of granular material is composed of a certain number of particle contacts, so the particle contact is a type of contact surface with a certain area. The void part in the void cell system of these six particles is shown in Figure 6. As shown in Figure 6 (left), if particles A and B are in contact and squeeze each other, the voids in the four void cells form a void homeomorphic to a ring [21]. As shown in Figure 6 (right), if the two particles are not in contact, the void parts in the void cells form a void homeomorphic to a sphere rather than a void homeomorphic to a ring (a body homeomorphic to a ring (sphere) means that there is one hole (no hole) inside it in topology). As shown by the red lines in Figure 5, in both cases the face $f_{1}^{\prime }$ formed by the closed edge cycle ($e_{1}^{\prime },\;e_{2}^{\prime },\;e_{3}^{\prime }$, and $e_{4}^{\prime }$) in the solid cell system intersects with the edge $e_{1}$ between particle A and particle B in the void cell system. If these two particles are in contact, the face $f_{1}^{\prime }$ characterizes a physical contact. If they are not in contact, it characterizes a virtual contact (the two parent particles in the two sides of the virtual contact are not in contact).

### 3.3. The Radical Voronoi Tessellation

In this paper, the radical Voronoi tessellation of the particle centers is chosen for constructing the solid cell system of the granular material made of spherical particles with different radii [22,23]. It consists of choosing the separation plane between two spheres in the radical plane, i.e., the points with equal tangents relative to the two spheres [22]. The solid cell system constructed by it has the property that if there is a contact point at a certain particle surface, this contact point will be on the face of the corresponding solid cell of this particle. 

The void cell system is constructed according to the duality relation between these two systems. In the solid cell system, each vertex composes four bodies. Therefore, in the void cell system, the body which the vertex in the solid cell system corresponds to is composed of four vertexes, which are the particle centers corresponding to these four bodies in the solid cell system.

**Figure 7:**
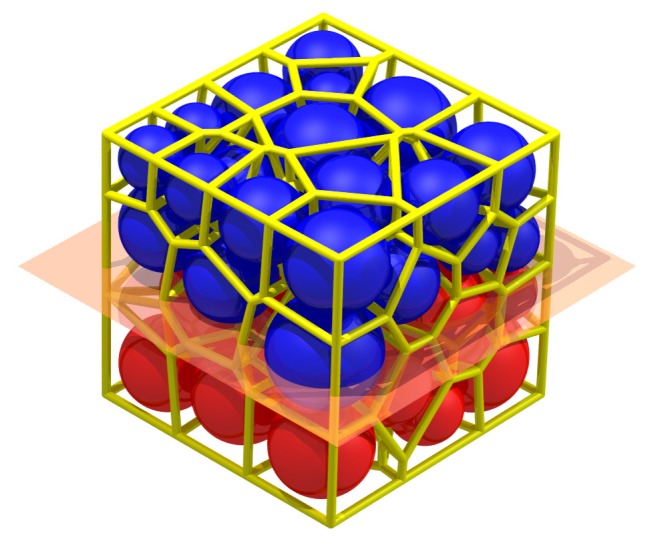
A typical spherical granular pack and its solid cell system.

Figure 7 shows a typical spherical granular pack and its solid cell system; each face of the system is the interface of two adjacent particles and can characterize the particle contact [21]. There is a presupposed surface in Figure 7. The particles above it are blue, while the particles below it are red.

### 3.4. The Role of the Particle, the Contact, and the Void

The correspondence between the composition of the granular material and the elements of the two dual geometric systems is shown in Table 1. The words in parentheses are the existence forms of the geometric systems.

### 3.5. The Characterization of the Particle Contact

In graph theory, for a graph G=(V, E), edge *e* is a pairs of vertex *v*:

(1) $$e=\left\{v_{i},\;v_{j}\right\}$$

A walk is an alternate sequence of vertices and edges of G of the form:

(2) $$w=\left(v_{i},\;\left\{v_{i},\;v_{j}\right\},\;v_{j},\;\left\{v_{j},\;v_{k}\right\},\;v_{k},\;\cdots ,\;v_{l},\;\left\{v_{l},\;v_{n}\right\},\;v_{n}\right)$$

A walk is termed a path if all of its vertices (and necessarily all of its edges) are distinct [24]. If, in the path, the starting vertex is the same as the ending vertex ($v_{i}=v_{n}$), the path is termed a cycle. A face can be described by the closed edges in its outer boundary, so the particle contact can be described by a cycle in the solid cell system $G^{\prime }=\left(V^{\prime },\;E^{\prime }\right)$. The detailed formula is shown in Equation (3):

(3) $$f^{\prime }=\left(v_{i}^{\prime },\;\left\{v_{i}^{\prime },\;v_{j}^{\prime }\right\},v_{j}^{\prime },\;\left\{v_{j}^{\prime },\;v_{k}^{\prime }\right\},\;v_{k}^{\prime },\;\cdots ,\;v_{l}^{\prime },\;\left\{v_{l}^{\prime },\;v_{i}^{\prime }\right\},\;v_{i}^{\prime }\right)$$

## 4. Characterization Method of the Macro Contact 

### 4.1. The Definition of the Macro Contact 

**Figure 8:**
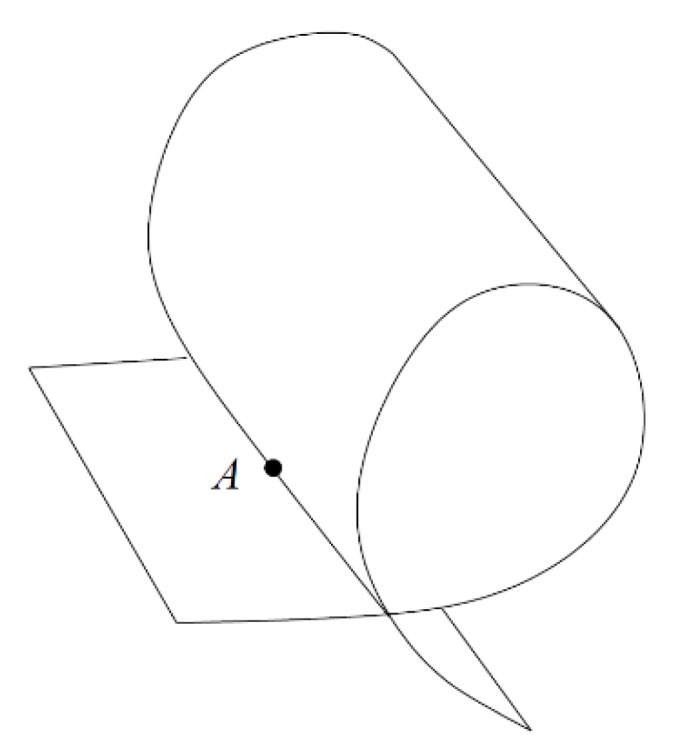
A surface with a self-intersection [26].

The following provides a clearer definition of the macro contact of granular material. The definition of a 2-manifold (two-dimensional manifold) is introduced. In topology, 2-manifolds are those topological spaces in which every point has a neighborhood that is topologically equivalent to an open disk [25]. A 2-manifold can be interpreted intuitively as a surface that does not intersect itself. Figure 8 is a typical example of a surface with self-intersection. Obviously, a macro contact surface should be locally homeomorphic to a disk at non-boundary regions, and a half-disk at boundaries. It should satisfy the restrictive condition of a 2-manifold with a boundary.

The definition of the macro contact can be stated with respect to two aspects: physics and topology. In physics, it is composed of a certain number of particle contacts. In topology, it is a 2-manifold with a boundary. Therefore, the macro contact is a particle contact set which satisfies the restrictive condition of a 2-manifold with a boundary.

### 4.2. The Characterization of the Macro Contact

The macro contact is a surface composed of a certain number of micro contacts. Each micro contact can be characterized by a face $f^{\prime }$ in the solid cell system. Therefore, the macro contact can be characterized by a face set $F^{\prime }$ which satisfies the constraint of a 2-mainfold with a boundary in the solid cell system.

The face $f^{\prime }$ can be treated as a type of two-dimensional polygonal element. Therefore, the face set $F^{\prime }$ which characterizes the macro contact can be treated as a two-dimensional mesh composed of these elements, and this mesh should be a 2-manifold at non-boundary regions.

A polygon mesh is a 2-manifold at non-boundary regions if it contains neither non-manifold edges, non-manifold vertices, nor self-intersections. The solid cell system constructed by radial Voronoi tessellation divides the space into non-overlapping polyhedrons [27,28]. Therefore, one face only intersects another face at its edges rather than at other regions in the solid cell system. For this reason, if there are no non-manifold edges and non-manifold vertices in the two-dimensional mesh composed of polygonal faces in the solid cell system, this mesh is a 2-manifold.

**Figure 9:**
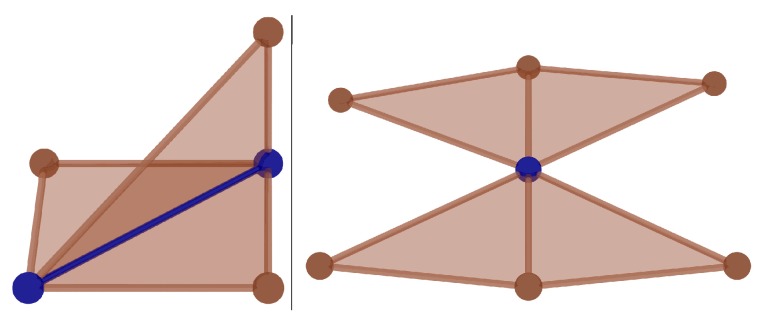
The example of non-2-manifold mesh (two surface sheets meet at a non-manifold vertex (**right**). A non-manifold edge has more than two incident faces (**left**)) [29].

A non-manifold edge has more than two incident polygons. A non-manifold vertex is generated by pinching two surface sheets together at that vertex such that the vertex is incident to more than one fan of polygons. Figure 9 shows an example of a non-2-manifold mesh [29].

In detail, we assume that there is a polygon face set $F=\left\{f_{1},f_{2},f_{3},\cdots f_{n}\right\}$. There is an edge set which is composed of the edges of all the faces in F:

(4) E={e∣e∈f,f∈F}

There is a vertex set V which is composed of the vertexes of all the faces in F:

(5) V={v∣v∈f,f∈F}

If F,E,V satisfy the following constraints, the faces in the face set F is a 2-manifold.

1. There are no non-manifold edges; no more than two polygons share an edge [26].

If there is an edge e such that $e\in f_{a}$, $e\in f_{b}$ ($f_{a}\in F,\;f_{b}\in F,f_{a}\neq f_{b}$), then $e\notin f_{c}$ ($\forall f_{c}\in F,f_{c}\neq f_{a},f_{c}\neq f_{b}$) [26].

2. There are no non-manifold vertices [26].

Consider the set of all polygons containing the vertex $v_{i}$. Consider the set of all edges of these polygons not containing $v_{i}$ itself [26]. This set is called the link of $v_{i}$ [26]. We assume that this set is finite, and if there is a permutation ($e_{1},e_{2},e_{3},\cdots e_{n}$) such that $e_{i}$ and $e_{i+1}$ share a vertex and no non-consecutive edges which do not share a vertex except, possibly, $e_{1}$ and $e_{n}$, vertex $v_{i}$ is a non-manifold vertex [26]. If $e_{1}$ and $e_{n}$ share a vertex, the vertex $v_{i}$ is called an internal vertex; otherwise, the vertex $v_{i}$ is called a boundary vertex [26]. 

**Figure 10:**
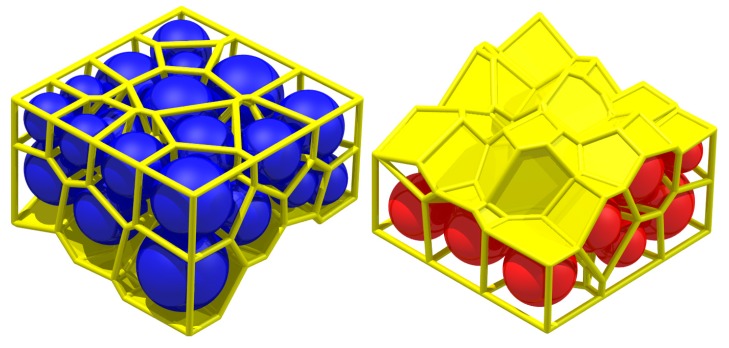
The face set which characterizes a macro contact of granular material.

The yellow faces in Figure 10 depict the face set which characterizes the presupposed macro contact of the granular pack in Figure 7. The face set is a macro contact (an interface) between the blue particles and red particles. Apparently, the face set is a 2-manifold at non-boundary regions because it contains neither non-manifold edges, non-manifold vertices, nor self-intersections at non-boundary regions.

## 5. The Application with DEM

### 5.1. A Brief Introduction to DEM

The discrete element method (DEM) is a numerical method which allows finite displacements and rotations of discrete bodies and new contacts between the blocks or particles which are automatically recognized and updated as the calculation progresses [30]. DEM can be adopted to simulate the mechanic behavior of soil and rock. This is because rock or soil can be treated as the agglomeration of different cemented mineral grains, which has been verified by numerous mesoscopic experimental tests.

In this study, we employed particle flow code (PFC), a commercial software that realizes the computational process of DEM. The macro cohesion of soil and rock can be simulated by the bonds between the particles in PFC. The principle of PFC has been extensively described in other papers [12], so this paper will briefly describe the key features of PFC that are used in this study [31].

A contact-stiffness model can provide a relation between the normal ($F_{n}$) and shear ($F_{s}$) components of contact forces and the relative displacements ($U_{n}$, $U_{s}$) of the particles. The simplest model is the linear model. It is assumed that, for two particles A and B in contact, their normal stiffness values are $K_{n}^{A}$ and $K_{n}^{B}$ and their shear stiffness values are $K_{s}^{A}$ and $K_{s}^{B}$, respectively, and the normal and shear stiffness of the contact can be computed by the following equations:

(6) $$K_{n}=\frac{K_{n}^{A}\cdot K_{n}^{B}}{K_{n}^{A}+K_{n}^{B}}$$

(7) $$K_{s}=\frac{K_{s}^{A}\cdot K_{s}^{B}}{K_{s}^{A}+K_{s}^{B}}$$

The force-displacement law of two particles in contact is as follows: $F_{n}$ is the normal contact force; $F_{s}$ is the shear contact force; $U_{n}$ is the normal overlap between the two particles in contact; $U_{s}$ is the tangential overlap.

(8) $$F_{n}=K_{n}\cdot U_{n}$$

(9) $$\Delta F_{s}=K_{s}\cdot \Delta U_{s}$$

**Figure 11:**
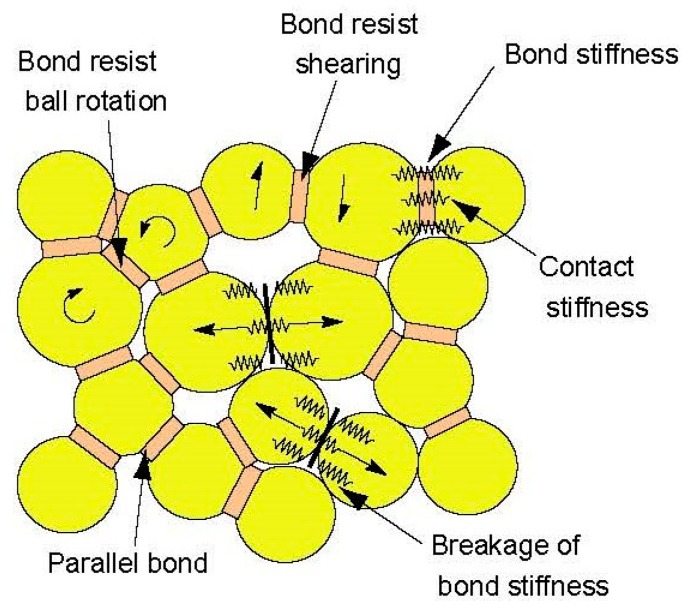
Illustration of parallel bond models provided in PFC (Particle Flow Code) [33].

As is shown in Figure 11, the parallel-bond contact model is adopted in this study to simulate the cemented particles. The maximum tensile and shear stresses acting on the bond periphery are calculated (via beam theory) to be [32]:

(10) $$\sigma_{\max}=\frac{-{\bar{F}}^{n}}{A}+\frac{\left|{\bar{M}}^{s}\right|}{I}\bar{R}$$

(11) $$\tau_{\max}=\frac{\left|{\bar{F}}^{s}\right|}{A}+\frac{\left|{\bar{M}}^{n}\right|}{J}\bar{R}$$

A is the area of the bond disk, J is the polar moment of inertia of the disk cross-section, and I is the moment of inertia of the disk cross-section about an axis through the contact point. $\bar{F}$ is the force at the bond. $\bar{M}$ is the moment at the bond [32]. n is the normal vector and s is the tangential vector. If the maximum tensile stress exceeds the normal strength or the maximum shear stress exceeds the shear strength, then the parallel bond breaks [32].

The equations of motion can be expressed as two vector equations. The equation for translational motion can be written in the vector form [32]:

(12) $$F_{i}=m\left(\ddot{x_{i}}+g_{i}\right)$$

where $F_{i}$ is the resultant force, the sum of all externally-applied forces acting on the particle; m is the total mass of the particle; and $g_{i}$ is the body force acceleration vector (e.g., gravity loading) [32]. The equation for rotational motion can be written in the vector form [32]:

(13) $$M_{i}=\dot{H_{i}}$$

where $M_{i}$ is the resultant moment acting on the particle and $\dot{H_{i}}$ is the angular momentum of the particle [32].

### 5.2. The Visualization of the Crack

As is mentioned in Section 2, if there are cracks within a cemented granular material, the cracks always appear between the adjacent mineral grains rather than inside a certain particle. Therefore, it is assumed that all the cracks appear between the adjacent particles in the computational process of DEM. When the particles in contact are judged to separate or slip, a micro-crack will appear at the corresponding particle contact. Similarly, a macro-crack can be treated as the separation or slip of the particles on the two sides of the macro-contact. Therefore, the crack and its corresponding contact have the same shape and can be characterized by the characterization method of the contact. For the rock, the macro strength is controlled by the bonds between the mineral particles. Therefore, the break of the bond is set as the index of the appearance of the crack on the contact. For example, as is shown in Figure 7, if the blue particles are cemented together and the red particles are cemented together while there are no bonds between the blue particles and red particles, the face set in Figure 10 is the macro-crack of this sample.

**Figure 12:**
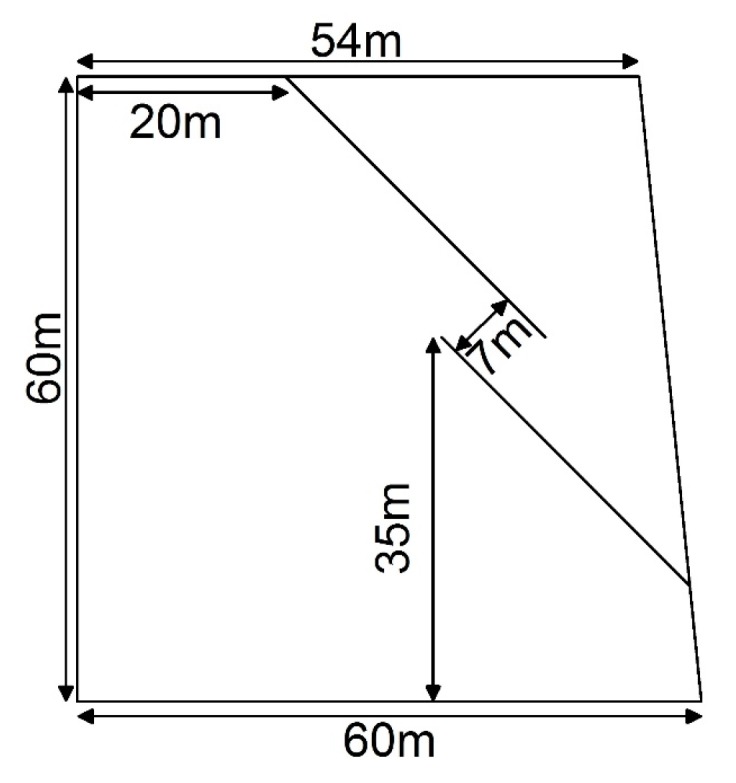
The section of the rock slope with parallel fractures and its size.

**Figure 13:**
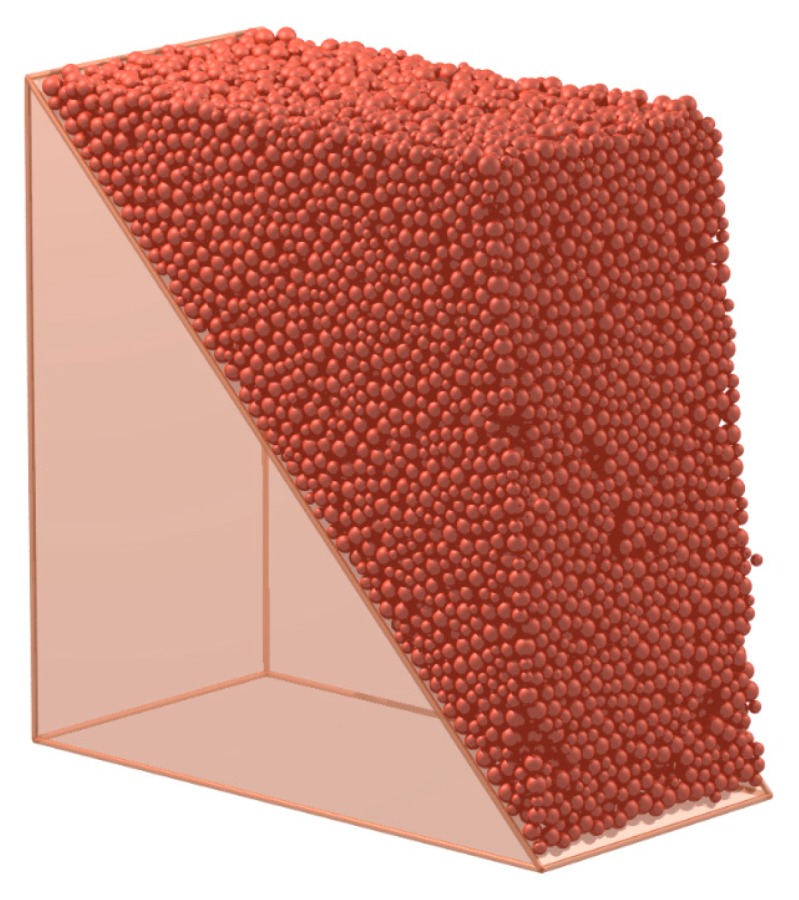
A rock slope simulated by DEM.

This study takes a fractured rock slope to show the detail of the visualization of the crack. A DEM model is built to simulate a rock slope with parallel fractures. Its width is 30 m and its section and size are shown in Figure 12. The model is constructed by the generation of the random particles inside the boundaries, as shown in Figure 13. For computational efficiency, some particles in the bottom are not constructed. The parameters of the example are shown in Table 2 and Table 3. The size of the particle is larger than the real size of the mineral grains, so the parameters of the particles are called mesoscopic parameters. The mesoscopic parameters are determined by the calibration in the virtual biaxial compression test in the DEM [34]. The gravity is $9.8\;m/s^{2}$; the timestep is 0.00024 s.

**Figure 14:**
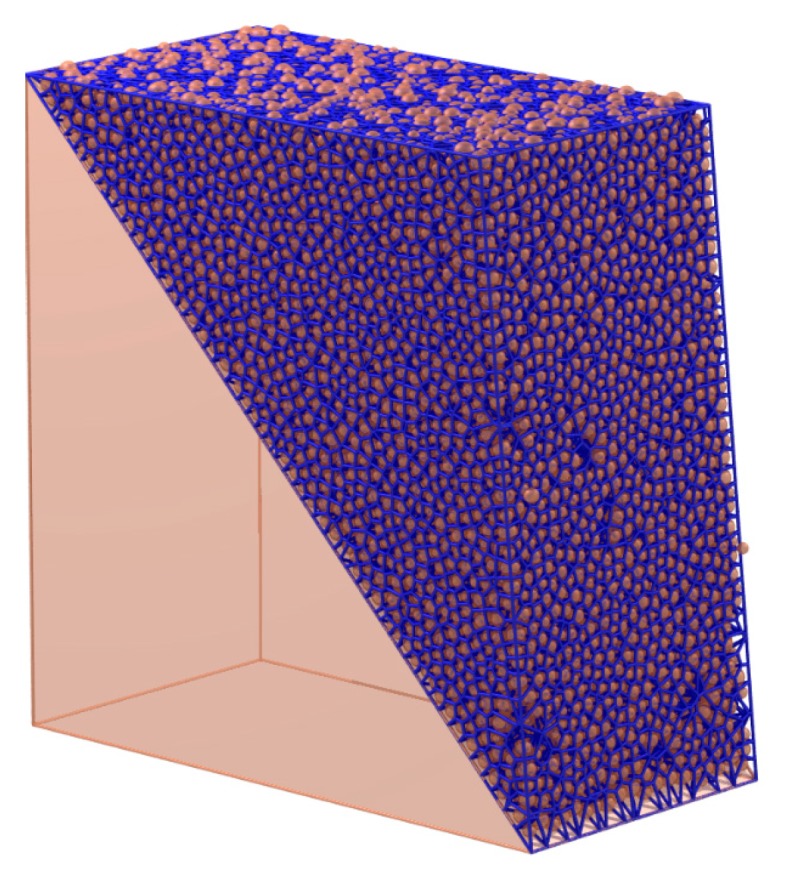
The solid cell system in State 1.

As is shown in Figure 14, the solid cell system is constructed by radical Voronoi tessellation. The particle contacts are characterized by the faces of the solid cell system. These faces can be classified into three groups: 

Group A1 (the virtual contact): If the distance of the two particles on the two sides of the face in the solid cell system is greater than the sum of the radii of the two particles, the two particles are not in contact physically. The object characterized by the face is set as a virtual contact.

Group A2 (the physical contact with a bond): If the distance of the two particles on the two sides of the face in the solid cell system is smaller than the sum of the radii of the two particles, the two particles are in contact physically. The object characterized by the face is a physical contact. If there is a bond on this contact, this contact is a physical contact with a bond.

Group A3 (the physical contact without a bond): If there is no bond on the physical contact, this contact is a physical contact without a bond.

For rocks, the bonds between the mineral particles are the main controlling factors of the macro strength. Therefore, Group A1 and Group A3 can be treated as potential weaknesses of the granular material. In this paper, they are set as the mesoscopic cracks of the rock. 

**Figure 15:**
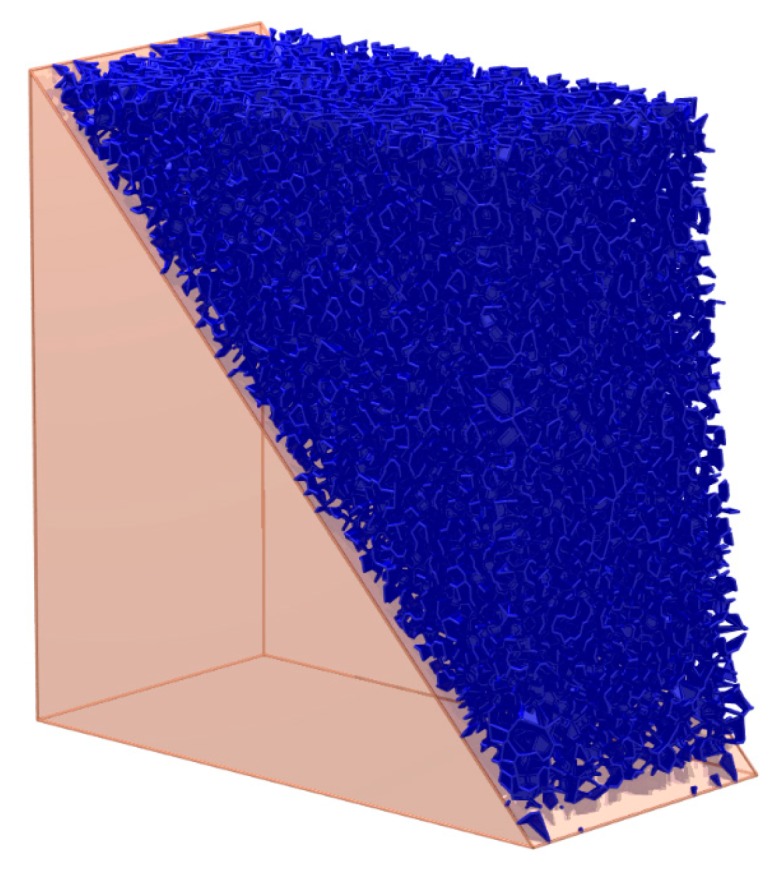
The mesoscopic cracks in State 1.

The mesoscopic cracks in the initial state are shown in Figure 15. This state is set as State 1. In State 1, there are some virtual contacts in the sample, which indicates that the particles at this place do not contact each other closely. There are no physical contacts without bonds for the reason that all the bonds have not been broken in State 1. The virtual contacts and the physical contacts without bonds in State 1 can be treated as the initial imperfections or mesoscopic cracks of the sample.

**Figure 16:**
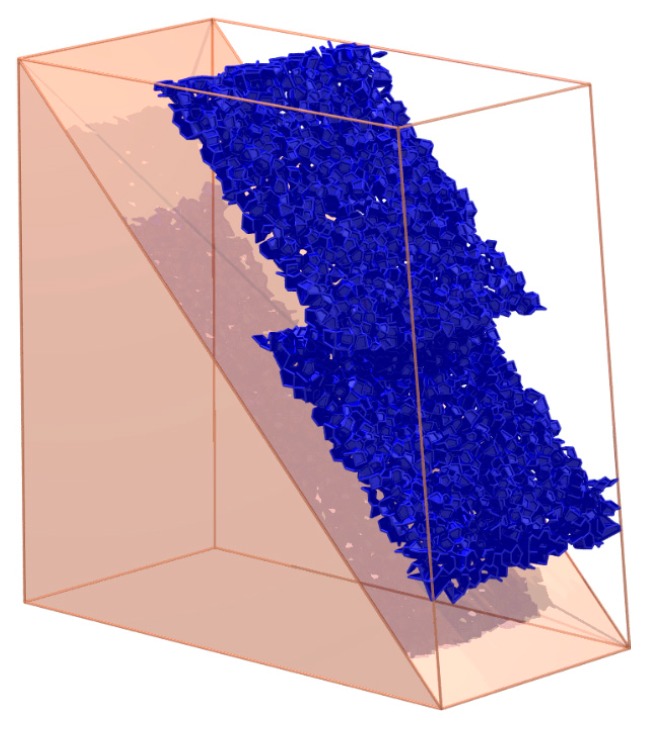
The bond is deleted to construct the parallel fracture in the example.

The slope is stable in State 1. Then, as shown in Figure 16, the parallel fractures are constructed by deleting the bonds at this place. The state after this construction is set as State 2. The mesoscopic cracks (the virtual contact and the physical contact without a bond) in State 2 can be classified into three groups:

Group B1: The cracks exist both in the initial State 1 and State 2.

Group B2: There is a bond in a certain contact; however, the bond breaks because of the loads, then the physical contact with a bond becomes a physical contact without a bond or a virtual contact, which leads to the appearance of the crack. This kind of crack is set as Group B2.

Group B3: There is no physical contact or virtual contact between a certain pair of particles, and the particles change their positions because of the loads. Then, the physical contact or virtual contact appears. This can be treated as a crack between this pair of particles. This kind of crack is set as Group B3.

Group B2 and Group B3 can be treated as the mesoscopic cracks caused by the loads.

### 5.3. The Determination of the Failure Route

Most rocks exhibit the characteristic of high brittleness. For these kinds of rocks, there will be an obvious major failure surface when it breaks under load. In DEM, this phenomenon appears as though the locations of the breaking bonds are centered in a surface. When the brittle failure occurs, the intact specimen will become two or more clusters divided by the failure surface (in DEM, the cluster is defined as a group of balls, all of which can be reached by traversing bonds).

In the void cell system, all the vertices and the edges characterizing the physical contacts with bonds can be extracted to form a new graph. Each vertex represents a particle. Its adjacent matrix A is the matrix in which the element value $a_{\mathrm{ij}}$ is 1 or 0, which means that there is or is not an edge between vertex $v_{i}$ and $v_{j}$, respectively. In physics, this means that there is or is not a physical contact with a bond between the particles characterized by $v_{i}$ and $v_{j}$. The adjacent matrix A is a sparse matrix because one particle is only in contact with a few particles in a granular sample, consequently saving a large amount of computing resources and time. The determination of the cluster is transformed to a mathematical problem: whether there is a path from one vertex to another vertex in this extracted graph. If there is a path formed by the contact with a bond, the two particles characterized by the corresponding vertexes belong to one cluster, which can be solved by graph theory [35].

The interface of two clusters can be solved by searching all the edges between the vertices characterizing different clusters in the void cell system. The faces in the solid cell system which correspond to these edges in the void cell system can present the shape of the failure surface between the two clusters.

**Figure 17:**
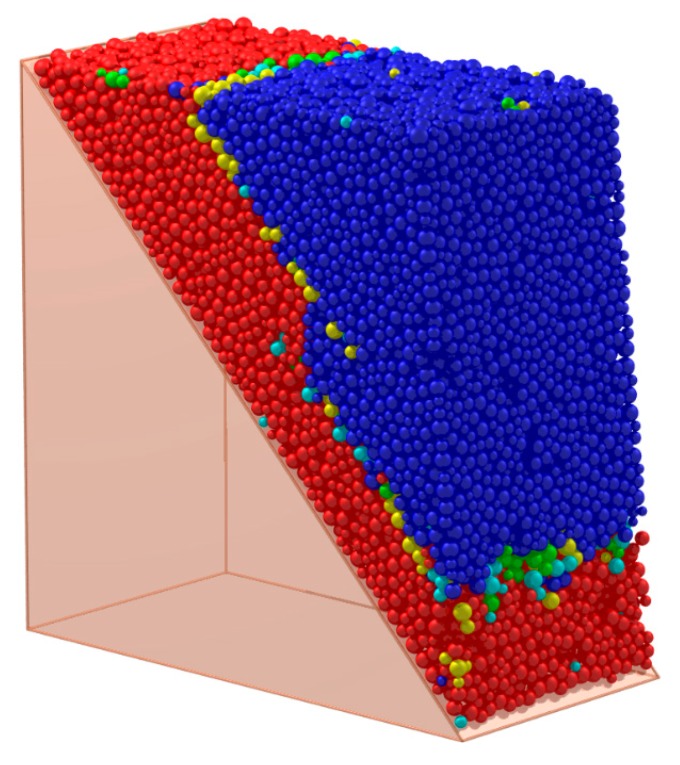
The clusters of the slope in state 2.

**Figure 18:**
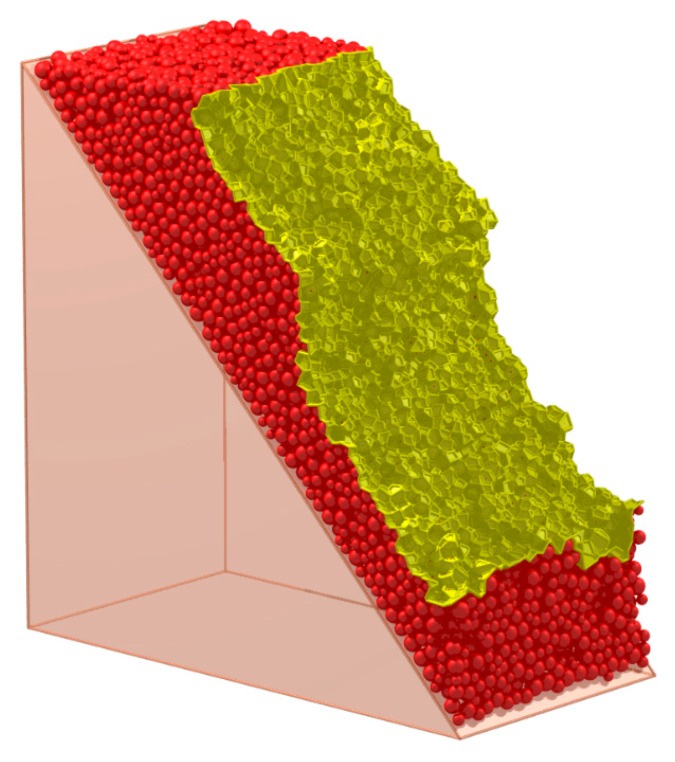
The interface between the bottom cluster and the other clusters.

In the example of Section 4.2, the clusters after 0.5 s of State 2 are shown in Figure 17. The result indicates that two major clusters appear. If other small clusters are merged into the two major clusters, the interface between the two clusters appears as shown in Figure 18. Above all, the characterization method in this paper can describe more details of the failure route in DEM; it can calculate the macro failure route from a micro perspective.

### 5.4. The Solution of Macro Contact Force

**Figure 19:**
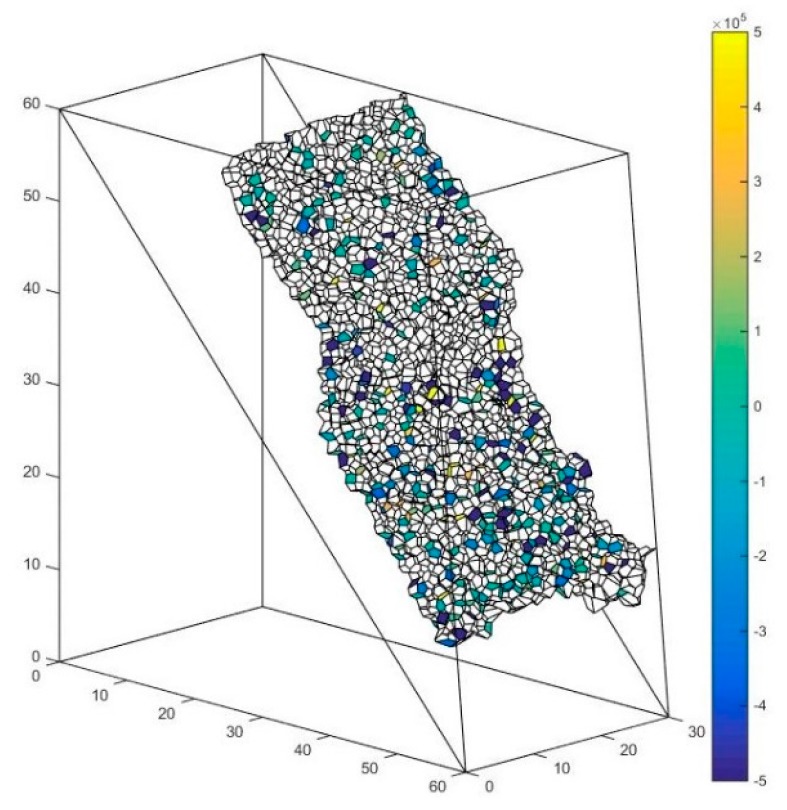
The contact force of the particle contact along the x-direction.

**Figure 20:**
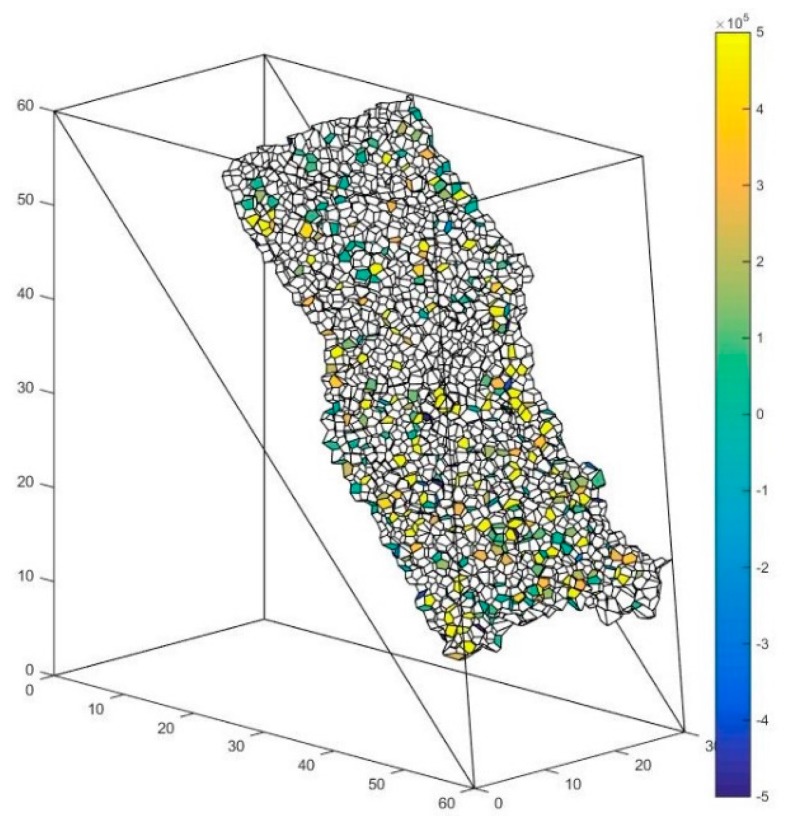
The contact force of the particle contact along the y-direction.

**Figure 21:**
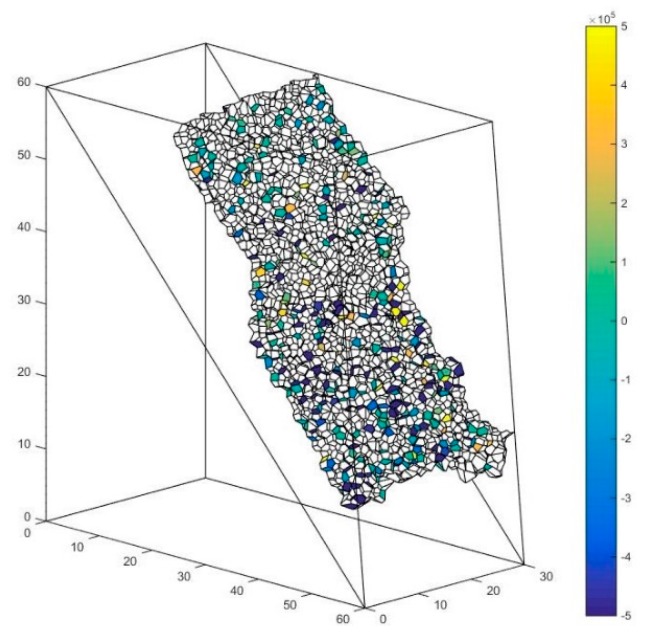
The contact force of the particle contact along the z-direction.

The abovementioned slope is taken as an example. As is shown in Figure 18, the interface between these major clusters is set as the presupposed contact surface. The contact forces of the particle contacts forming the macro contact are shown in Figure 19, Figure 20 and Figure 21 respectively. The white faces in these figures are in virtual contact, which means that the particles on the two sides of the face are only adjacent, but not in physical contact. Therefore, there are no contact forces in these faces. The result shows that the contact forces of the macro contact in DEM are absolutely discrete, which is different from the result obtained using a continuation method. This method can help obtain the result comparable to the result of a continuation method such as FEM (Finite Element Method).

$f^{\prime }$ is defined as the face in the solid system used to characterize the particle contact. Every face $f^{\prime }$ can be assigned to a weight $w\left(f^{\prime }\right)$ to describe the contact force of $f^{\prime }$. $F^{\prime }$ is defined as the face set characterizing the macro contact of the granular material. $W\left(F^{\prime }\right)$ is set as the contact force of the macro contact characterized by the face set $F^{\prime }$. Since the force vectors can be added, $W\left(F^{\prime }\right)$ can be calculated by adding the contact force of the particle contact in the macro contact:

(14) $$W\left(F^{\prime }\right)=\underset{f^{\prime }\epsilon F^{\prime }}{\sum }w\left(f^{\prime }\right)$$

### 5.5. Summary

Section 5 shows how the geometrical changes, forces, and cracks of a macroscopic contact surface in a cemented particulate material are tracked by contact characterization. The parameters of the sample are calibrated, but have nothing to do with some of the actual examples. The accuracy of the simulation results depends on the constitutive model and the boundary conditions, but this is not the purpose of this paper.

The effect of the characterization method of the contact is to help follow the geometrical changes of the simulated results. Researchers have turned to complicated three-dimensional numerical simulations in order to investigate more characteristics of granular materials. The lack of an analytical method may lead to the shortage of descriptions of simulated results. For example, the three-dimensional DEM analysis of the step-path failure in jointed rock slopes was discussed recently [36,37], but most of the analysis of the simulated result was restricted to a two-dimensional cross-section analysis. The characterization method of the contact can help to elucidate more details of the three-dimensional failure mode.

## 6. Conclusions

This paper proposed a characterization method of micro-macro contacts of three-dimensional granular materials made of spherical particles, particularly the cemented granular material made of spherical particles. It has a clear physical basis and is quantifiable in mathematics. On the macro level, the particle contact is a type of surface contact with voids in its surrounding rather than a point contact. The macro contact is a particle contact set satisfying the constraint of a 2-manifold with a boundary.

In terms of the mathematical characterization, the two dual geometrical systems are abstracted from the granular pack. The face in the solid cell system is extracted to characterize the particle contact. The face set satisfying the constraint of a 2-manifold with a boundary characterizes the macro contact. With a clear physical basis and simple mathematical operability, this characterization can integrate the originally-discrete particle contacts with continuous macro contacts, bridging the gap between the mesoscopic and macro scales of granular materials.

DEM is a type of discontinuous mechanic method which simulates the mesoscopic property of granular materials. This paper improves the post-processing of DEM. For example, this paper raises the visualization method of micro-macro cracks. The quantified failure surface can be acquired by the algorithms in graph theory. In addition, it puts forward a method to calculate the macro-micro contact force in DEM from a micro perspective, which can obtain the macro contact force of any macro contact surfaces. 

## Figures and Tables

**Table 1:** The correspondence between the composition of the granular material and the elements of the two dual geometric systems.

-	Void Cell System G	Solid Cell System $G^{\prime }$
**Particle**	Vertex	body
	(particle center)	(particle cell)
**Contact**	Edge	Face
	(particle connection)	(particle contact surface)
**Seepage path**	Face	Edge
	(seepage path section)	(seepage pipe)
**Void**	Body	Vertex
	(void cell)	(void center)

**Table 2:** The mesoscopic parameters of the example.

Mesoscopic Parameters	Values
Particle density $\rho_{p}$	2000 kg/m^3^
Particle radius R	0.5 m–1.0 m
Normal contact stiffness $K_{n}$	5 × 10^8^ N/m
Shear contact stiffness $K_{s}$	5 × 10^8^ N/m
Friction coefficient of the particle surface $\mu_{p}$	0.1
Normal parallel bond stiffness ${\bar{k}}_{n}$	5 × 10^8^ N/m
Shear parallel bond stiffness$\;{\bar{k}}_{s}$	5 × 10^8^ N/m
Normal parallel bond strength NBS	4.5 × 10^6^ Pa
Shear parallel bond strength SBS	3.5 × 10^6^ Pa

**Table 3:** The macro parameters of the example.

Marco Parameters	Values
Soil unit weight γ	17 kN/m^3^
Cohesion c	500 kPa
Internal friction angle φ	15°
Elastic modulus $E^{\prime \prime }$	300 MPa
Poisson’s ratio ν	0.2
